# Supplementary material for: Endosomal microautophagy is activated by specific cellular stresses in trout hepatocytes
Source: Sci Rep. 2025 Nov 10;15:39347. doi: 10.1038/s41598-025-23022-x (PMC12603132; doi:10.1038/s41598-025-23022-x)
Supplement: Supplementary file 1 — Supplementary Material 1 [file 41598_2025_23022_MOESM1_ESM.pdf]

Endosomal Microautophagy is Activated by Specific Cellular Stresses in Trout Hepatocytes

Emilio J. Vélez[1, 2, *, #], Vincent Véron[1, #], Jeanne Gouis[1], Steffi Reji[1], Karine Dias[1], Amaury Herpin[3], Florian Beaumatin[1], Iban Seiliez[1, *]

[1]INRAE, Université de Pau et des Pays de l'Adour, UMR1419 Nutrition Métabolisme et Aquaculture, Saint-Pée-sur-Nivelle, France, 64310.

[2]Present address: Department of Cell Biology, Physiology and Immunology, Faculty of Biology, University of Barcelona, Barcelona, Spain.

[3]INRAE, UR1037 Laboratory of Fish Physiology and Genomics, Rennes, France, 35700.

*Corresponding authors: **Iban SEILIEZ** and **Emilio J. VELEZ**, INRAE, UMR1419 NuMeA, 64310 Saint-Pée-sur-Nivelle, France. iban.seiliez@inrae.fr; evelezve@ub.edu

[#]Equal contribution.

Supplementary Material

- **Fig. S1.** The core eMI machinery is present and expressed in *Danio rerio* (zebrafish) tissues.
- **Fig. S2.** The silencing of the ESCRT-I protein Tsg101 by using si*tsg101* did not prevented the formation of CMA puncta induced by mild-oxidative stress exposure, while the morpholino-mediated knockdown of the Tsg101 interacting protein chaperone Bag6 prevented the formation of eMI puncta.
- **Fig. S3.** Uncropped images of the immunoblots shown in **Figures 3C**, **Figure 3G**, and **Supplementary Figure 2G**.

A

Gene name	*Homo sapiens*		*Danio rerio*	
	**ID-gene**	**Chr**	**ID-gene**	**Chr**
TSG101	ENSG00000074319	11	ENSDARG00000040854	25
			ENSDARG00000011897	7
VPS4a	ENSG00000132612	16	ENSDARG00000030114	25
VPS4b	ENSG00000119541	18	ENSDARG00000069175	2
ALIX	ENSG00000170248	3	ENSDARG00000025269	19
BAG6	ENSG00000204463	6	ENSDARG00000075892	15
			ENSDARG00000077531	19

B

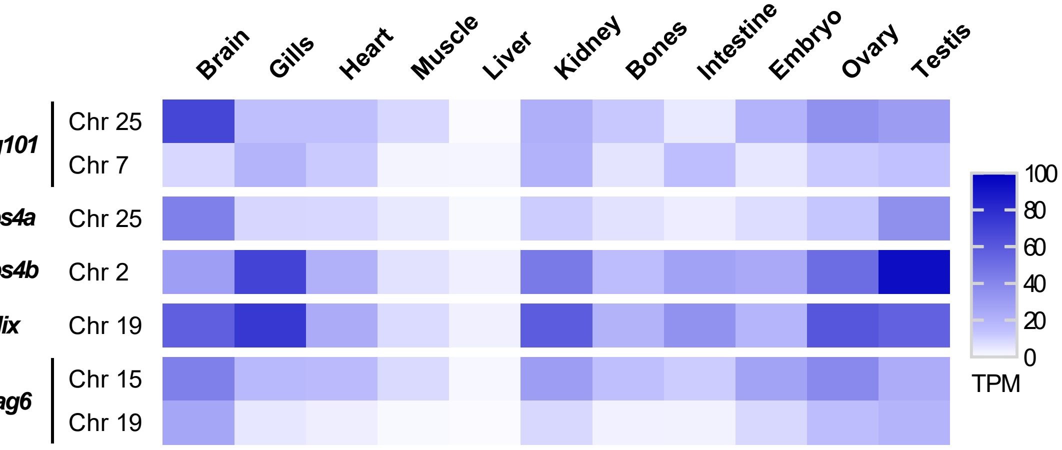

Supplementary Figure 1. **The core eMI machinery is present and expressed in *Danio rerio* (zebrafish) tissues**. (**A**) Identification (Ensembl ID) and chromosome location (Chr) of the main eMI-genes (*TSG101*, *VPS4A*, *VPS4B*, *ALIX* and *BAG6*) in the human genome, and their corresponding zebrafish orthologs. (**B**) Heat map showing mRNA expression levels (TPM, transcripts per million) of the different identified genes in different zebrafish tissues (brain, gills, heart, muscle, liver, kidney, bones, intestine, embryo, ovary and testis) extracted from the RNA-Seq database PhyloFish.

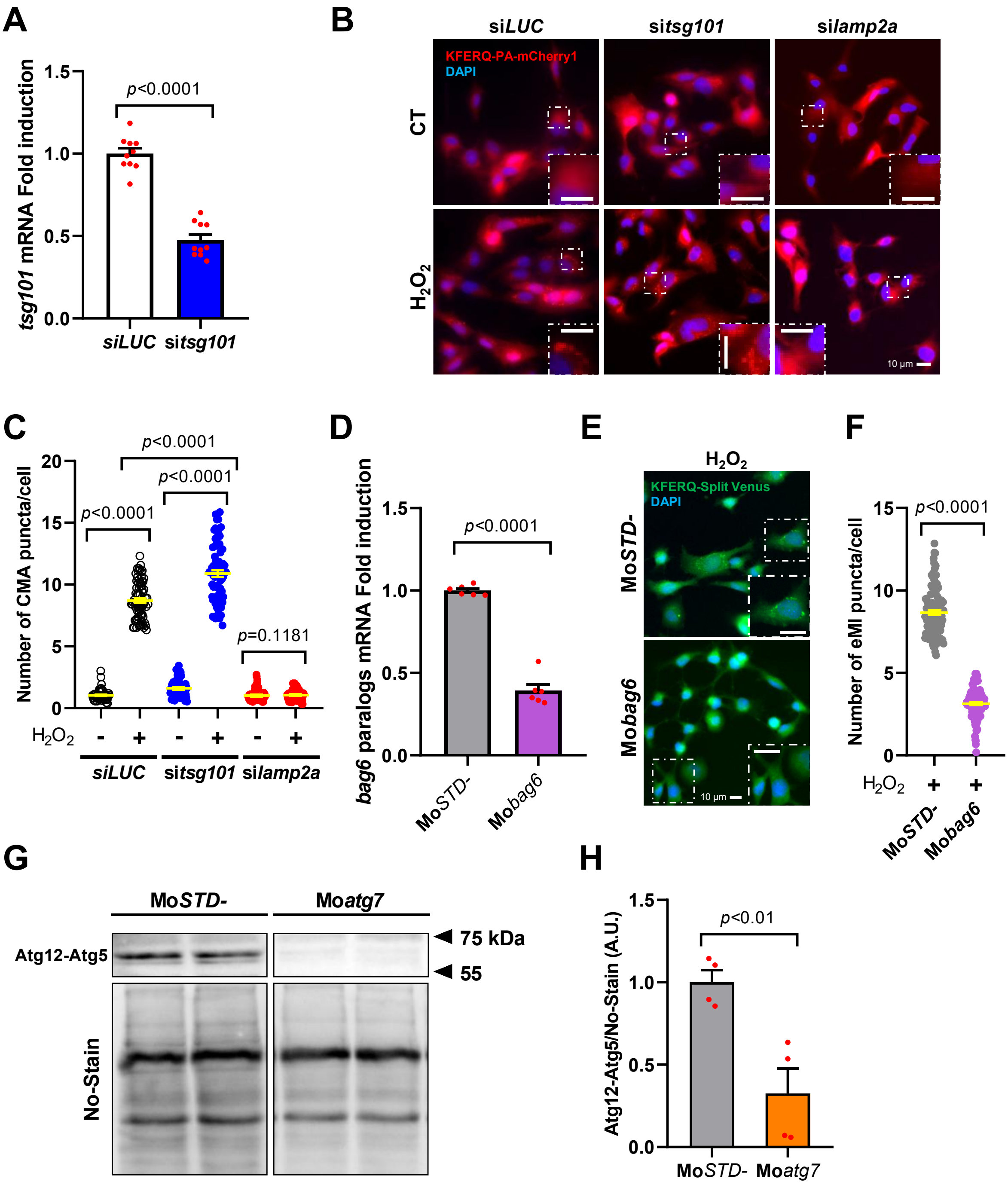

Supplementary Figure 2. **The silencing of the ESCRT-I protein Tsg101 by using si*tsg101* did not prevented the formation of CMA puncta induced by mild-oxidative stress exposure, while the morpholino-mediated knockdown of the Tsg101 interacting protein chaperone Bag6 prevented the formation of eMI puncta.** (**A**) The *tsg101* mRNA levels were significantly downregulated by the transfection of RTH-149 cells with si*tsg101*. Differences with respect to the negative control condition (si*LUC*) were assessed using Unpaired Student's T-test ($p < 0.0001$) in five independent experiments with duplicates. (**B**) Representative images of RTH-149 cells

expressing the KFERQ-PA-mCherry1 CMA-reporter and transfected with either negative control siRNA (si*LUC*), si*tsg101*, or a combination of two siRNAs targeting the two *lamp2a* paralogs (si*lamp2a*) of RT, and incubated with CT medium or exposed to mild-oxidative stress (H_2O_2 25 µM) for 16 h, and the (**C**) quantification of CMA number of puncta per cell. All values correspond to individual images (si*LUC* CT 60; si*LUC* H_2O_2 77; si*tsg101* CT 65; si*tsg101* H_2O_2 70; si*lamp2a* CT 60; si*lamp2a* H_2O_2 55), with >20 images/experiment in a total of 3 independent experiments (> 600 cells per condition). Differences between two groups were assessed using the non-parametric Mann Whitney test, and the p-values are indicated in the figure. (**D**) The mRNA levels of all four RT bag6 paralogs were significantly reduced by the transfection of RTH-149 cells with a Mo*bag6* cocktail. Differences with respect to the standard negative control condition (Mo*STD-*) were assessed using Unpaired Student's T-test ($p < 0.0001$) in three independent experiments with duplicates. (**E**) Representative images of RTH-149 cells expressing the eMI-reporter transfected with either Mo*STD-* or Mo*bag6* and exposed to H_2O_2 25 µM for 16 h, and (**F**) quantification of KFERQ-Venus number of puncta per cell. All values correspond to individual images (Mo*STD-* 112; Mo*bag6* 106), with >32 images/experiment in a total of 3 independent experiments (> 1310 cells per condition). Differences between the two groups were assessed using Mann Whitney test ($p < 0.0001$). (**G**) Representative image of western blot against Atg5 in RTH-149 cells transfected with Mo*atg7* or a negative control (Mo*STD-*), exposed during 16 h to H_2O_2 25 µM, and (**H**) quantification of Atg12-Atg5 complex protein levels normalized to No-Stain. Differences between groups were assessed using parametric Unpaired Student's T-test ($p = 0.0070$) in four single independent experiments. All data are presented as Mean ± SEM; scale bars: 10 µm.

Figure 3C

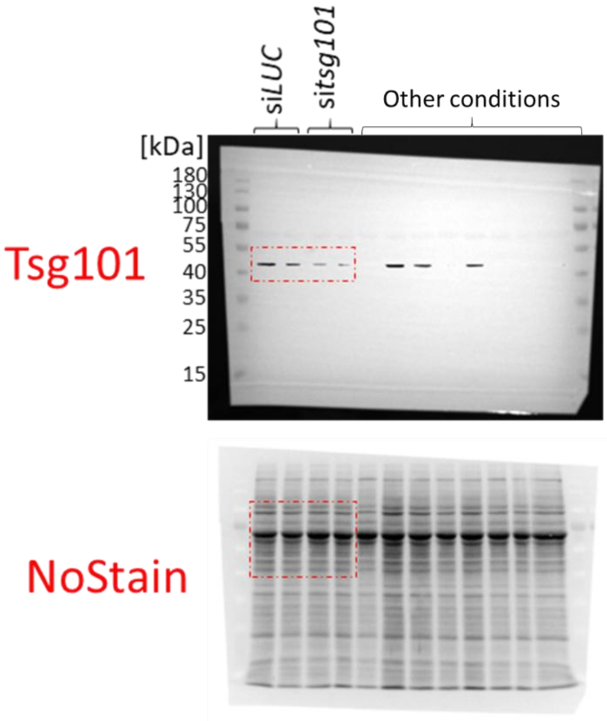

Figure 3G

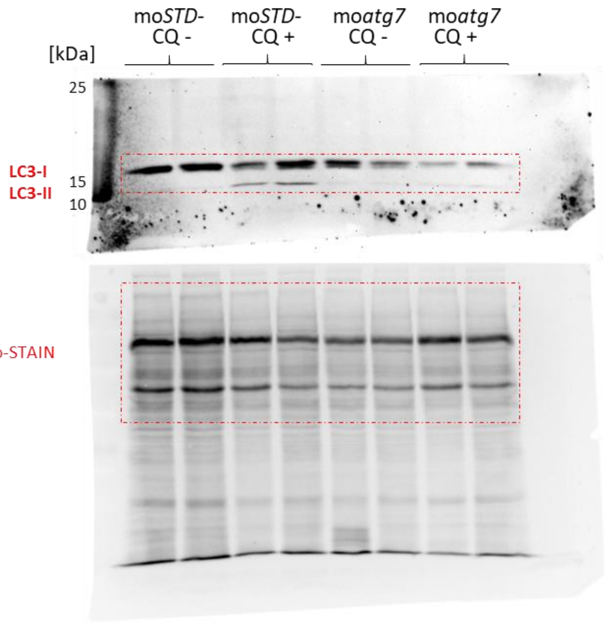

Figure Supplementary 2G

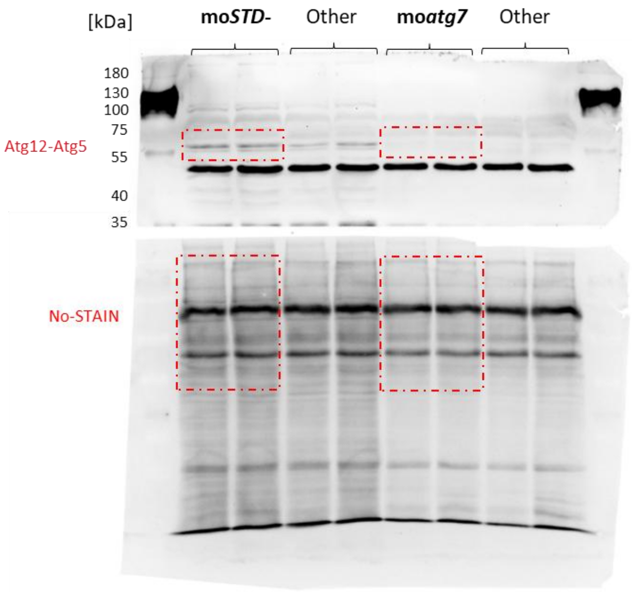

Supplementary Figure 3. Uncropped images of the immunoblots shown in Figures 3C, Figure 3G, and Supplementary Figure 2G. The red box marks the portion of the blots shown in the figures.
